# Triple Threat or Mere Inconvenience? Exploring the Effect of COVID-19 Precautions, Lack of Access to Lactation Care, and the Infant Formula Shortage on Breastfeeding Behavior of Parents in the Midwest of the United States

**DOI:** 10.1007/s10995-026-04237-4

**Published:** 2026-02-25

**Authors:** Elizabeth Kar, Lisa Akers, Savanna Westrom

**Affiliations:** 1 North Dakota State University, EML Hall 316 F, NDSU Dept 2620, P.O. Box 6050, Fargo, ND 58108–6050 USA; 2 Center for Nutrition and Health Impact, Omaha, USA; 3 University of Minnesota, Duluth, USA

**Keywords:** Pandemic, Formula shortage, Lactation consultant, Breastfeeding, Duration

## Abstract

**Objective:**

To explore the combined effects of COVID-19 and the infant formula shortage.

**Methods:**

A cross-sectional survey explored breastfeeding behavior by COVID-19 infant feeding guidance, access to lactation support, and awareness and effect of the infant formula shortage. A multilinear regression model with backward elimination with bootstrapping was used to determine statistically significant predictors of breastfeeding duration. Text responses were analyzed to calculate the number and type of effects of the infant formula shortage.

**Results:**

The number of effects of the infant formula shortage on the family, access to lactation support in the hospital, and number of hours of employment outside the home were the three most predictive variables for breastfeeding duration. However, access to lactation support was the only predictor that was statistically significant. Free form text responses demonstrated that formula feeding and some breastfeeding parents felt stress from the infant formula shortage.

**Discussion:**

Access to lactation support in the hospital is widely recognized as a predictor of breastfeeding success. The decrease in lactation support that occurred during the COVID-19 pandemic may have created a barrier to breastfeeding. It is also remarkable that, regardless of feeding practice, the infant formula shortage created stress for families.

**Conclusions for Practice:**

Access to a lactation consultant continues to be a significant contributor to breastfeeding behavior. Infant formula shortages may create risks for formula fed and breastfed infants. Breastfeeding protection and promotion may be a critical step in decreasing dependence on infant formula.

**Supplementary Information:**

The online version contains supplementary material available at 10.1007/s10995-026-04237-4.

## Significance

Research has considered the isolated impact of COVID-19 safety measures and the infant formula shortage on breastfeeding behavior. However, there is little research on the combined effects of these crises on breastfeeding behavior. This study considered three simultaneous threats, or a triple threat, to breastfeeding duration among a sample of parents in the United States (U.S.). Threats included the COVID-19 safety guidance, lack of access to lactation care, and the infant formula shortage. Understanding the combined effects of these events will be critical in planning for future emergencies.

**Supplementary Information:**

The online version contains supplementary material available at 10.1007/s10995-026-04237-4.

## Introduction

During the last 15 months of the COVID-19 pandemic, the U.S. experienced an infant formula shortage from which it is mostly recovered (U.S. Government Accountability Office, 2025). While COVID-19 is no longer classified as a public health emergency and is considered an on-going health issue, or endemic, exploring its effects on families is still critical in preparing for future outbreaks (World Health Organization, 2023). Additionally, understanding the combined effects of co-occuring crises, like the formula shortage, is also of value in future planning.

### COVID-19 Pandemic

The impact of the COVID-19 pandemic on breastfeeding duration varied depending on when in the pandemic the data was gathered. During 2020, among a sample of 1344 hospitals participating the Maternity Practices in Infant Nutrition and Care (mPINC) survey, 68.9% (924) facilities reported no change in their exclusive breastfeeding rates during hospital admission, while 11.3% and 12.3% experienced an increase or decrease in exclusivity rates, respectively (Perrine et al., 2020). In a survey of pregnant women about their perception of the safety and adequacy of their prenatal care as it related to provision of services early in the pandemic, only 3.1% of the 258 respondents indicated that the pandemic influenced their infant feeding decision (Burgess et al., 2021). Another 1.6% of respondents indicated they might change their infant feeding plan to include breastfeeding because of concerns about the infant formula supply and to better protect their baby (Burgess et al., 2021). Finally, in another study examining breastfeeding rates among participants in the Supplemental Nutrition Program for Women, Infants and Children (WIC) in Los Angeles, California, the percentage of infants receiving breastmilk at 3 and 6 months decreased by 8% and 10% respectively from the prepandemic time period to 2022 (Koleilat et al., 2022).

Reasons for changes in breastfeeding rates during COVID-19 are multifaceted. Factors that may have reduced breastfeeding rates included forced separation of mother and infant in the hospital to prevent COVID-19 transmission (Popofsky et al., 2020) and lack of access to qualified lactation care (Koleilat et al., 2022; Perrine et al., 2020). However, some changes during the COVID-19 pandemic may have increased breastfeeding rates in some groups. These include breastfeeding to provide immune protection (Koleilat et al., 2022), increase in remote work options (Pritz et al., 2024), visitor restriction policies in hospitals (Perrine et al., 2020), and concerns about lack of access to infant formula due to hording and supply chain disruptions (Imboden et al., 2023).

### Infant Formula Shortage

Prior to May 5, 2023, when World Health Organization declaring that the COVID-19 pandemic was no longer a global public health emergency, a major U.S. formula manufacturer recalled multiple products in their infant formula line due to microbial contamination and subsequently closed a production plant in February 2022 (Doherty et al., 2022; United States Food & Drug Administration, 2023). This created two contiguous events. At the time of the plant closure, there were already supply chain issues limiting the availability of goods, including infant formula (United States Food & Drug Administration, 2023). The result was a severe shortage of infant formula. The U.S. Food and Drug Administration (FDA) employed multiple strategies to alleviate the shortage, which focused on strengthening the formula supply chain (United States Food & Drug Administration, 2023). However, there has been little discussion of decreasing reliance on infant formula through breastfeeding support and promotion (United States Food & Drug Administration, 2023).

Some evidence suggests the formula shortage improved breastfeeding initiation and duration rates (Cernioglo & Smilowitz, 2023; Imboden et al., 2023; Seoane Estruel & Andreyeva, 2025). A review of medical records of newborn, 1 month, and 2-month-old infants at a rural pediatric care office showed significant increases in breastfeeding initiation, and at 1 and 2 months from the pre-shortage to the shortage sample (Imboden et al., 2023). While initiation rates were only slightly higher (approximately 2%), the 1- and 2-month rates were approximately 8% higher in the shortage group (Imboden et al., 2023). The differences were statistically significant. However, while breastfeeding rates were higher in some communities, not all families were able to access breastfeeding to cope with the formula shortage. In a cross-sectional survey of 99 parents in the U.S., 75% of which were WIC participants, approximately 48.5% of respondents used one “unsafe” infant feeding practice during the formula shortage (Cernioglo & Smilowitz, 2023). Unsafe feeding practices were defined as overdiluting infant formula or using homemade or expired infant formula. Additionally, the researchers found that use of donor milk, and informal breastmilk sharing increased, while the use of mother’s own milk decreased (Cernioglo & Smilowitz, 2023).

### Combined Effects

While research has explored, in isolation, the effects of COVID-19 and the infant formula shortage on breastfeeding initiation and duration, there is little research that explores the combined effects of these crises. Infants and young children are among the most vulnerable populations during natural and man-made emergent situations, and families in crisis may need more support to feed their children adequately and safely (Bilgin & Karabayır, 2024; World Health Organization & United Nations Children’s Emergency Fund, 2003). Given the challenges families may face in feeding their children safely during one crisis level event, it is important to understand the impact of two occurring simultaneously.

### Objectives

The specific objectives for this paper are to identify the effect on breastfeeding behavior of the following variables: (1) COVID-19 guidance for safe infant feeding; (2) availability of lactation resources after delivery; (3) the infant formula shortage; and (4) determine if there was any combined effect of all three of these on infant feeding practices posing a triple threat to families of infants. To our knowledge, there is no other research exploring the relationships between the three variables listed.

## Methods

**Fig. 1:**
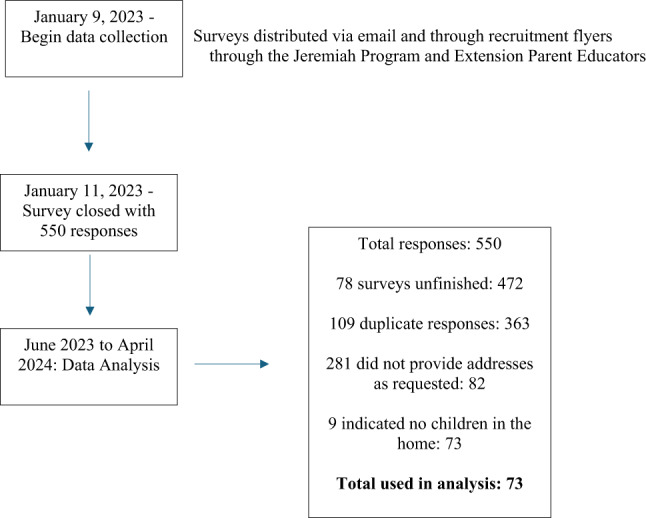
Timeline for data collection and number of responses

This project was approved by the Institutional Review Board at North Dakota State University protocol #HE21050. The study was performed in accordance with the ethical standards established in the 1964 Declaration of Helsinki and its subsequent amendments. The study was a cross-sectional survey. The study was funded by the principal investigator’s employing institution. The full timeline and number of participants are listed in Fig. 1.

### Instrument

The survey instrument was adapted from an instrument used in a previous study (Brotherson, 2024). The instrument used in the previous study was not validated. The survey was not pilot test prior to administering. The adapted version of the instrument used in the present study included six items from the previous instrument (Brotherson, 2024), 14 items assessing demographic characteristics, four from the Pregnancy Risk Assessment and Monitoring Survey (PRAMS) (Centers for Disease Control and Prevention, 2023; Hilliard et al., 2022), and four original items. Demographic information collected included: if the participant had a child born between 2019 and 2023, participant’s and child’s ages and genders, participant’s and child’s race and ethnicity, household size, household income, and employment status. To assess breastfeeding behavior, the instrument included seven questions about breastfeeding behavior and support. These included: prenatal infant feeding plan and social support for that plan; education provided on safe infant feeding practices to limit COVID-19 transmission and if that information influenced the infant feeding plan; if the infant was still breastfeeding, age of infant when breastfeeding ceased; and if breastfeeding support in the hospital was available. Finally, there were three questions measuring awareness and effect of the infant formula shortage. The last 13 questions focused on child development concerns and use of telehealth. The entire survey instrument included 38 total questions. Most questions were multiple choice and included a write in response option for “other”. There were two fully open-ended write in questions. The survey was formatted using Qualtrics (Provo, UT,^ 2020^). Researchers tested the instrument for functionality before launching.

### Participants

The target population was adults over age 18 who delivered a child between December 2019 and January 2023. Participants were recruited from two vastly different groups in order to increase the likelihood of having a more diverse sample. The two programs were the state extension Parent Education program and the local Jeremiah Program.

The Parent Education program is administered through state extension agencies with the purpose of providing evidence-based resources to facilitate effective parenting (North Dakota State University Extension, 2026). Programming is administered through Extension Parent Educators and includes activities that facilitate nurturing behavior between parents and children, seminars on parenting topics, and Parent Cafés where parents meet for support (North Dakota State University Extension, 2026). Demographics of the program participants during 2022–2023 were 78% white, 59.4% married or partnered, and 37.5% with incomes at $75,000 or more per year (blinded for review). Participant ages were not given.

The Jeremiah Program is designed to provide support to single mothers who intend to pursue a college education (Jeremiah Program, n.d.). To be eligible for the program, the single mother must be 18 years of age, have at least 50% custody of a child under the age of five, plan to attend college in the next six months, and attend programing on the Jeremiah Program campus (Jeremiah Program, n.d.). In addition to mentorship, coaching, and leadership training, the program also provides childcare stipends and educational programming for children (Jeremiah Program, n.d.). Participants in the local Jeremiah Program mostly identify as white (44%), Black (26%), or two or more races (14%) (A. Klein, personal communication, May 20, 2024). Additionally, they all have incomes under $36,000 per year with 88% under $20,000 per year. The majority have a high school education (68%).

Parent Educators emailed all parent participants the recruitment flyer which contained a QR code to the survey. The flyer described the eligibility criteria and advertised a $15 Walmart gift card incentive for survey completion. Upon scanning the QR code, participants were taken to the survey. The first part of the survey included the informed consent document and a question asking participants to confirm their consent to participate before allowing them to proceed with the survey. Jeremiah Program participants accessed the QR code for the survey through the recruiting flyer which was posted at the facility. They gave consent in the same manner as the other group. To receive the $15 Walmart gift card, parents had to provide an address to which the incentive could be mailed. The informed consent document and the survey stated explicitly that an address must be provided because accounting restrictions at the parent institution required this. Participants were informed that the address was only for mailing and would not be shared.

### Analysis

Demographic data was assessed with descriptive statistics. Multilinear regression modeling with backward elimination identified predictive factors for breastfeeding duration. The dependent variable was breastfeeding duration, which was measured by the age of the child when breastmilk was no longer provided or by the child’s current age if the child was “still breastfeeding.” There were various predictive (independent) variables measured including demographic data, infant feeding intention, whether COVID-19 infant feeding guidance was provided, access to a lactation consultant, and awareness and effects of the infant formula shortage. Categorical variables were coded as either dummy, ordinal or scaled variables for assessment. Coding is included at the bottom of Table 3. Backward elimination was chosen to identify significant predictors of breastfeeding duration. All analyses were preformed using IBM SPSS Statistics (version 29). Assumptions for normality of residuals, homoscedasticity, linearity, and absence of multicollinearity were verified. All assumptions were met except for homoscedasticity. This is likely due to the addition of new questions that were not previously pilot tested. The sample used in the multilinear regression analysis was composed of the 29 participants who had children under the age of one year, as they were the only ones who received the infant formula shortage questions. Bootstrapping was applied to each of the eight models generated through multilinear backward regression to mitigate problems with heteroscedasticity due to small sample size (Tabachnick & Fidell, 2019).

## Results

Figure 1 highlights the timeline and number of surveys submitted and selected for analyses. Five hundred fifty surveys were submitted. Many surveys were either incomplete or duplicates. To ensure that only unique completed surveys were used for analysis, the researcher chose to analyze only those that were fully complete and provided a unique mailing address. This left 82 surveys. Nine of those respondents indicated no children in the home. The final sample included 73 surveys (13%). Table 1 displays participant demographic information. Participants were more likely to identify as white (94–96%), have an income greater $75,000 per year (54–57%), reside in North Dakota (76–88%), and be employed outside the home full-time (54–57%). A notable exception was those who were still breastfeeding were more likely to be employed part-time (24%) or not working outside the home (24%).

**Table 1:** Participant demographics for the entire sample and divided by still breastfeeding or not

Demographic	Total participant (%) (*n*=73)	No longer BF^a^ (%) (*n*=56)	Still BF^a^ (%) (*n*=17)
Parent race			
Asian	1 (1.4)	1 (1.8)	0 (0.0)
Black	1 (1.4)	1 (1.8)	0 (0.0)
Black and white	1 (1.4)	0 (0.0)	1 (5.9)
White	69 (94.4)	53 (94.6)	16 (94.1)
Other: Mexican American	1 (1.4)	1 (1.8)	0 (0.0)
Child race (per parent)			
AN^b^/AI^c^ and Black	1 (1.4)	1 (1.8)	0 (0.0)
AN^b^/AI^c^ and NHPI^d^	1 (1.4)	1 (1.8)	0 (0.0)
Black	1 (1.4)	0 (0.0)	1(5.9)
Black and NHPI^d^	1 (1.4)	1 (1.8)	0 (0.0)
Black and white	7 (9.6)	6 (10.7)	1(5.9)
White	62 (84.8)	47 (83.9)	15 (88.2)
Parent Hispanic or Latin X			
Hispanic	4 (5.4%)	2 (3.6)	2 (11.8)
Puerto Rican	1 (1.4%)	1 (1.8)	0 (0.0)
Spanish	1 (1.4%)	1 (1.8)	0 (0.0)
Latin X	1 (1.4%)	1 (1.8)	0 (0.0)
Didn’t specify	1 (1.4%)	1 (1.8)	0 (0.0)
No	65 (89.0)	50 (89.2)	15 (88.2)
Household income			
<$15,000	7 (9.6)	4 (7.2)	3 (17.6)
$15,000–$24,999	2 (2.8)	2 (3.6)	0 (0.0)
$25,000–$34,999	2 (2.8)	2 (3.6)	0 (0.0)
$35,000–$49,999	10 (13.7)	8 (14.3)	2 (11.8)
$50,000–$74,999	11 (15.1)	8 (14.3)	3 (17.6)
$75,000–$99,999	21 (28.7)	15 (26.7)	6 (35.4)
>$100,000	20 (27.3)	17 (30.3)	3 (17.6)
Household composition			
Total number in home			
2	5 (6.8)	4 (7.1)	1 (5.9)
3	22 (30.2)	18 (32.1)	4 (23.5)
4	25 (34.2)	16 (28.6)	9 (52.9)
5	12 (16.4)	9 (16.1)	3 (17.7)
6	6 (8.2)	6 (10.7)	0 (0.0)
7	1 (1.4)	1 (1.8)	0 (0.0)
8	1 (1.4)	1 (1.8)	0 (0.0)
11	1 (1.4)	1 (1.8)	0 (0.0)
Total adults			
1	8 (11.0)	6 (10.7)	2 (11.8)
2	61 (83.4)	46 (82.1)	15 (88.2)
3	2 (2.8)	2 (3.6)	0 (0.0)
4	2 (2.8)	2 (3.6)	0 (0.0)
Total children			
1	29 (39.7)	24 (42.9)	5 (29.4)
2	23 (31.5)	15 (26.7)	8 (47.1)
3	13 (17.8)	9 (16.1)	4 (23.5)
4	5 (6.8)	5 (8.9)	0 (0.0)
5	1 (1.4)	1 (1.8)	0 (0.0)
6	1 (1.4)	1 (1.8)	0 (0.0)
9	1 (1.4)	1 (1.8)	0 (0.0)
State of residence			
North Dakota	62 (84.8)	49 (87.5)	13 (76.4)
Minnesota	8 (11.0)	6 (10.7)	2 (11.8)
New York	1 (1.4)	0 (0.0)	1 (5.9)
Wisconsin	1 (1.4)	0 (0.0)	1 (5.9)
South Dakota	1 (1.4)	1 (1.8)	0 (0)
Employment status			
Not employed outside the home	9 (12.3)	5 (8.9)	4 (23.5)
Currently seeking employment	3 (4.1)	2 (3.6)	1 (5.9)
Employed, working < 25 h/week	10 (13.7)	6 (10.7)	4 (23.5)
Employed, working 25–39 h/week	12 (16.4)	11 (19.7)	1 (5.9)
Employed, working > 40 h/week	39 (53.3)	32 (57.1)	7 (41.2)

^a^BF = breastfeeding

^b^AN = Alaskan Native

^c^AI = American Indian

^d^NHPI = Native Hawaiian/Pacific Islander

Table 2 lists response frequencies and breastfeeding duration for questions related to the infant formula shortage. Individual responses for these questions are reported separately because data regarding the effect of the formula shortage on the family was collected in free-form text. Most respondents were aware of the formula shortage (86.2%), and indicated that it did not affect their infant feeding decision (70.0%). However, free form text responses indicated that some breastfeeding families felt added stress about the need to maintain breastfeeding because of the formula shortage (*n* = 7, 10%).

**Table 2:** Infant formula shortage and effect on infant feeding practices (*n* = 73)

Question stem and responses	Total survey *n* (%)	BF completed *n* (%)	BF (mo)
Are you aware of the current infant formula shortage? (only answered if indicated they had a child < 12 months of age)			
Yes	25 (34.2)	11 (73.3)	2.07
No	4 (5.50)	4 (26.7)	6.28
Not given the question	44 (60.3)		
Did the formula shortage affect how you chose to feed your baby?			
No	20 (27.4)	9 (60.0)	2.97
Made me want to breastfeed	9 (12.3)	6 (40.0)	3.52
Made me want to use formula	0 (0.0)	0 (0.0)	
Other	0 (0.0)	0 (0.0)	
Not given the question	44 (60.3)	0 (0.0)	
What effect did the infant formula shortage have on your family? (participants could select more than 1)			
Pumped breastmilk to build a surplus	1	NA	NA
Increased breastfeeding intention	2		
Increased stress over breastmilk supply	7		
Increased formula expense for family	3		
Caused infant formula change	2		
Stockpiled infant formula	1		
Increased effort to find formula	6		
Concern for loved ones using formula	1		
No effect	13		
Breastfeeding duration by number of effects of formula shortage on family.			
0 effects	11 (37.9)	4 (26.7)	4.50
1 effect	14 (48.3)	7 (46.7)	2.53
2 effects	3 (10.3)	3 (20.0)	4.00
3 effects	1 (3.4)	1 (3.4)	0.12

Multilinear regression modeling with backward elimination using bootstrapping demonstrated that the three factors most predictive of breastfeeding duration while controlling for all other variables were the number of effects the family felt from the infant formula shortage, whether the breastfeeding parent had a lactation consultant visit while in the hospital, and number of hours worked outside the home (Table 3, Model 6) [F (3, 25) = 6.30, *p* =.00, R^2^ = 0.43, CI = 0.20, 0.66]. While the model is significant and explained 43% of the variance, the only significant predictor of breastfeeding duration was access to a lactation consultant. Therefore, the number of effects from the formula shortage and hours worked outside the home were removed from the final model, which included only access to a lactation consultant (Table 3, Model 8) [F (1,71) = 11.86, *p* <.00, R^2^ = 0.14, CI = 0.00, 0.26]. Race, income, awareness of the formula shortage, prenatal breastfeeding intention, and whether guidance on safe infant feeding during COVID-19 were not statistically significant as predictors.

**Table 3:** Backward multiple linear regression with bootstrapping and breastfeeding duration^a^ as the outcome measure (*n*=29)

Model				95%CI β					Model fit	
	Variables	B	SE	LL	UL	*p*	ΔR^2^	*R* ^2^	F_df_	*p*
1	Constant	−3.792	6.78	−17.42	9.45	0.57	N/A	0.50	2.48_8,20_	0.05
	Intention^b^	−1.13	5.20	−12.16	8.15	0.80				
	Income^c^	0.85	0.96	−1.17	2.71	0.36				
	Effects^d^	−4.71	2.66	−10.31	0.27	0.09				
	Race^e^	7.76	3.79	1.21	15.86	0.14				
	LC^f^	6.02	2.77	0.90	11.59	0.05				
	Aware^g^	0.45	3.22	−7.37	6.34	0.87				
	Employed^h^	−2.73	1.41	−5.25	0.18	0.10				
	COVID^i^	2.09	3.86	−5.58	9.86	0.56				
2	Constant	−2.49	6.02	−13.49	11.15	0.68	0.01	0.49	2.85_7,21_	0.03
	Intention^b^	−2.25	4.49	−12.75	5.87	0.54				
	Income^c^	0.75	0.93	−1.55	2.37	0.36				
	Effects^d^	−0.4.77	2.70	−10.20	0.48	0.08				
	Race^e^	7.11	3.67	0.39	14.94	0.15				
	LC^f^	6.62	2.69	1.65	12.41	0.04				
	Employed^h^	−2.66	1.31	−5.26	−0.06	0.07				
	Aware^g^	1.23	3.10	−5.49	7.36	0.58				
3	Constant	−0.90	4.35	−9.83	8.54	0.85	0.0	0.49	3.45_6,22_	0.02
	Income^c^	0.71	0.87	−1.13	2.11	0.39				
	Effects^d^	−5.03	2.49	−10.31	−0.33	0.06				
	Race^e^	7.25	3.45	1.26	14.71	0.12				
	LC^f^	6.79	2.39	2.31	11.40	0.03				
	Employed^h^	−2.67	1.27	−5.10	−0.00	0.08				
	Intention^b^	−2.79	3.83	−11.75	3.29	0.42				
4	Constant	−2.23	3.92	−9.54	6.49	0.60	0.01	0.48	4.22_5,23_	0.01
	Income^c^	0.78	0.83	−1.05	2.21	0.33				
	Effects^d^	−4.70	2.13	−9.26	−0.45	0.04				
	LC^f^	5.96	1.60	2.65	9.06	0.01				
	Employed^h^	−2.68	1.24	−5.02	−0.18	0.06				
	Race^e^	6.71	2.91	1.14	12.26	0.10				
5	Constant	4.04	3.52	−2.30	12.14	0.21	0.02	0.46	5.10_4,24_	0.00
	Effects^d^	−4.42	2.06	−8.81	−0.26	0.06				
	LC^f^	5.70	1.55	2.53	8.58	0.01				
	Employed^h^	−2.88	1.26	−5.27	−0.32	0.06				
	Income^c^	0.94	0.81	−0.74	2.50	0.26				
6	Constant	6.86	2.66	1.63	12.18	0.03	0.03	0.43	6.30_3,25_	0.00
	Effects^d^	−3.95	1.86	−7.85	−0.38	0.06				
	LC^f^	5.70	1.55	2.61	8.65	0.02				
	Employed^h^	−2.37	1.16	−4.59	−0.07	0.08				
7	Constant	2.50	1.37	−0.18	5.13	0.07	0.13	0.30	5.65_2,26_	0.01
	LC^f^	5.23	1.57	2.61	8.73	0.02				
	Effects^d^	−3.32	1.86	−7.14	0.25	0.09				
8	Constant	2.76	1.10	0.81	5.05	0.01	0.16	0.14	11.86_1,71_	<0.00
	LC^f^	3.01	0.82	1.37	4.64	<0.00				

^a^Breastfeeding duration was measured by the age of the child when the parent stopped providing breastmilk or the current age of the child if the parent is still providing breastmilk

^b^Breastfeeding intention – 0 = no intention, 1 = intention for any breastfeeding

^c^Family Income – 0 = < $15,000, 1 = $15,000–24,999, 2 = $25,000–34,999, 3 = $35,000–49,999, 4 = $50,000–74,999, 5 = $75,000–99,999, 6 = >$100,000

^d^Number of effects from infant formula shortage – 0 = no effects on family, 1 = 1 effect on the family, 2 = 2 effects on the family, 3 = 3 effects on family

^e^Parent race – 0 = White, 1 = other race

^f^Access to lactation support in the hospital – 0 = no support, 1 = support from someone other than and lactation consultant, 2 = support from a lactation consultant

^g^Aware of infant formula shortage – 0 = no, 1 = yes

^h^Employment outside the home – 0 = no, 1 = ≤ 25 h per week, 2 = 26–39 h per week, 3 = ≥ 40 h per week

^i^Told how to feed baby safely during COVID – 0 = no, 1 = yes

## Discussion

The results of this study demonstrate that the infant formula shortage caused stress not only in formula feeding families, but also in some breastfeeding families. Most research has focused, and rightly so, on the negative impact of the infant formula shortage on formula feeding families. The findings from this study are consistent with other studies in finding that formula feeding families felt strained by added expense from price gouging, “panic hoarding”, having to spend more time and effort finding formula, and having to switch brands to keep their child fed (Burgess et al., 2021; Channell Doig, 2024; Kalaitzandonakes et al., 2023). However, this study is unique in that breastfeeding families also had a platform to indicate that the infant formula industry and formula shortage are more far reaching than previously documented.

Whether the family received guidance on how to feed their infant safely during the COVID-19 pandemic did not seem to have a significant impact on breastfeeding duration. However, COVID-19 may have still influenced infant feeding practices. Previous studies (Burgess et al., 2021; Imboden et al., 2023) agree with our findings, that when parents’ infant feeding decision were influenced by the COVID-19 pandemic, they were usually skewed toward breastfeeding. The finding is not surprising given the immune benefits of breastfeeding, increased remote work options, and known infant formula supply chain issues that were present during this time.

There did not appear to be any combined effects of infant feeding guidance given during COVID-19, decreased access to lactation care, and the infant formula shortage. When all factors were included in multilinear regression model, the only significant predictor of breastfeeding duration was access to a lactation consultant. Our results are consistent with the body of literature (Patel & Patel, 2016) showing the beneficial effects of care from a lactation consultant on breastfeeding outcomes. This could also be an indication that limiting access to lactation consultants during a pandemic may have the unintended consequence decreasing breastfeeding duration, leaving infants exposed to illness.

### Limitations

While there was a large response rate to the survey and the sampling plan attempted to capture more participants of diversity, the majority of responses were disqualified because of duplication and incompletion. This left a sample of those who were where white, employed, and had a higher income than expected. With the rise of bots, survey research is getting more difficult to conduct using digitals means (Ball, 2019). The survey was not piloted before administering. Participants were offered a small incentive for participation ($15 Wal-Mart gift card). The ethics of incentives in health promotion research are not globally agreed upon (Vinay & Bauer, 2022). However, in this situation the researcher felt that compensating participants for their time was beneficial and did not promote coercion or inducement because of the small amount offered. Whether incentives should be offered moving forward should be considered carefully.

## Conclusions for Practice

The strongest predictor of breastfeeding duration was access to a lactation consultant. The pandemic and formula shortage had little combined effect on breastfeeding duration in multilinear regression modeling. Additionally, formula feeding families were not the only ones to feel stress from the formula shortage. Breastfeeding families do not live in a vacuum protected from the infant formula industry. Future research should explore in more depth the interaction between infant formula supply and breastfeeding behavior. Without regulation of formula manufacturers and additional investment in the protection and promotion of breastfeeding, all infants are at risk of harm during infant formula shortages.

## Supplementary Information

Below is the link to the electronic supplementary material.

Supplementary Material 1

## Data Availability

Please contact the corresponding author.
